# A Molecular Mechanism to Explain the Nickel-Induced Changes in Protamine-like Proteins and Their DNA Binding Affecting Sperm Chromatin in *Mytilus galloprovincialis*: An In Vitro Study

**DOI:** 10.3390/biom13030520

**Published:** 2023-03-12

**Authors:** Gelsomina Carbone, Gennaro Lettieri, Carmela Marinaro, Martina Costabile, Rosaria Notariale, Anna Rita Bianchi, Anna De Maio, Marina Piscopo

**Affiliations:** 1 Department of Biology, University of Naples Federico II, Via Cinthia, 21, 80126 Naples, Italy; 2 Department of Precision Medicine, School of Medicine, University of Campania “Luigi Vanvitelli”, Via Luigi de Crecchio, 80138 Naples, Italy

**Keywords:** nickel, spermatozoa, protamine-like, PARP, sperm chromatin, DNA, *Mytilus galloprovincialis*, nickel molecular mechanisms

## Abstract

Nickel is associated with reproductive toxicity, but little is known about the molecular mechanisms of nickel-induced effects on sperm chromatin and protamine-like proteins (PLs). In the present work, we analyzed PLs from *Mytilus galloprovincialis* by urea-acetic acid polyacrylamide gel electrophoresis (AU-PAGE) and SDS-PAGE and assessed their binding to DNA by Electrophoretic Mobility Shift Assay (EMSA) after exposing mussels to 5, 15, and 35 µM NiCl_2_ for 24 h. In addition, a time course of digestion with MNase and release of PLs from sperm nuclei by the NaCl gradient was performed. For all exposure doses, in AU-PAGE, there was an additional migrating band between PL-III and PL-IV, corresponding to a fraction of PLs in the form of peptides detected by SDS-PAGE. Alterations in DNA binding of PLs were observed by EMSA after exposure to 5 and 15 µM NiCl_2_, while, at all NiCl_2_ doses, increased accessibility of MNase to sperm chromatin was found. The latter was particularly relevant at 15 µM NiCl_2_, a dose at which increased release of PLII and PLIII from sperm nuclei and the highest value of nickel accumulated in the gonads were also found. Finally, at all exposure doses, there was also an increase in PARP expression, but especially at 5 µM NiCl_2_. A possible molecular mechanism for the toxic reproductive effects of nickel in *Mytilus galloprovincialis* is discussed.

## 1. Introduction

Anthropogenic activities such as industry, urbanization, and land development (agricultural and mining) have serious effects on the environment, with constant intake of pollutants in terrestrial and marine compartment [1,2,3,4]. The heavy metals represent one of the most significant environment pollutants [2,4,5,6,7,8,9,10,11], and their negative effects on human and animal health are widely studied in the literature [6,7,12,13,14]. A major problem of heavy metals is their ability to bioaccumulate in the environment, animal, and plants and, through the food chain, to reach humans [12,13,15]. One of the most studied heavy metals is nickel (Ni), particularly in industrialized countries, where it causes allergy problems through skin contact [13]. In this regard, the European union is implementing several directives to reduce the use and spread of nickel in the environment [16]. Problems caused by nickel include respiratory [17,18,19,20,21,22], liver, and kidney toxicity [22,23,24,25,26,27] and carcinogenic effects [28,29,30]. Human reproductive activity is also affected by the toxic action of nickel through the alteration of hormones [31]. Nickel also represents a problem in other environmental compartments, such as the marine environment [1] where it also negatively affects the reproductive health of marine organisms such as *Diadema savignyi* and *A. pulchella* as reported in Gissi et al., 2016 [1]. In addition, nickel in the coastal environment can have detrimental effects on the reproduction of copepods [32,33] and on ascidian sperm [34]. Given that reproduction is the basis of species survival, we investigated the possible effect of nickel on the reproductive health of one of the most-used organisms for the biomonitoring program, the Mediterranean mussel, *M. galloprovincialis. M. galloprovincialis* is an ideal model organism [5,35,36,37] because it is a filter-feeding sessile organism and accumulates different pollutants [7,36], such as heavy metals, hydrocarbons, and pesticides [5,7]. In addition, *M. galloprovincialis* is particularly abundant in the Mediterranean Sea [5,36,38] and thus represents an extremely important food source for populations living on the Mediterranean Sea coast [39,40]. *M. galloprovincialis* sperm chromatin is mainly organized by three protamine-like (PL) proteins (PL-II, PL-III, and PL-IV) [41]. PL proteins represent a structurally and functionally intermediate group of proteins between the histone (H) and protamine (P) type [42], belonging to Sperm Nuclear Basic Proteins (SNBPs)—chromosomal proteins associated with DNA in sperm nuclei at the end of spermiogenesis. PL proteins are arginine- and lysine-rich and are related to histone H1 [43,44]. In *M. galloprovincialis*, PL proteins represent 76% of all the SNBPs in the following proportions: PL-II (20%), PL-III (50%), and PL-IV (6%). These proteins coexist with about 20% of somatic histones and 4% of non-histones proteins [45]. In our previous work, we studied *M. galloprovincialis’* sperm chromatin organization, finding that sperm DNA is wrapped around a PL-III protein core and core histones, and PL-II and PL-IV are bound to the flanking DNA regions (similarly to somatic histone H1) [46]. In our previous papers, we already demonstrated the negative effects of some heavy metals, such as mercury, chromium, copper, and cadmium, on the reproductive health of *M. galloprovincialis* [5,7,35,36,37]. Therefore, in the present work we focused on evaluating the effects of nickel on the sperm chromatin and PL proteins’ properties of *M. galloprovincialis* after acute (24 h) exposure to three doses: 5, 15, and 35 µM of NiCl_2_. In the Mediterranean sea, nickel concentration is approximately between 3 and 4.5 nM on the surface and exceeds 5 nM in the Adriatic and Aegean Seas [47]. So, the doses used in this work mimic a long exposure of mussels to nickel. After exposure, first of all, we measured the nickel accumulated in the mussel gonads. Next, we analyzed, by SDS-PAGE and Acetic Acid-Urea Polyacrylamide gel (AU-PAGE), the electrophoretic pattern of the Protamine-like (PL) proteins and assessed their DNA-binding ability by electrophoretic mobility shift assay (EMSA). Finally, gonadal expression of PARP was evaluated, and the possible changes in sperm chromatin were studied by the release of PL proteins from sperm nuclei and preforming the time course of digestion with micrococcal nuclease (MNase).

## 2. Materials and Methods

### 2.1. Ethics Statement

This research was performed on the marine invertebrate *M. galloprovincialis* (Lamarck, 1819), which is not protected by any environmental agency in Italy. This study was conducted in strict accordance with European (Directive 2010/63) and Italian (Legislative Decree n. 116/1992) legislation on the care and use of animals for scientific purposes.

### 2.2. Bioaccumulation of Nickel

The analysis was carried out on tissues from 5 control mussels and exposed mussels. DigiBlock, a sample preparation technology, was used to digest the samples (LabTech, MA, Hopkinton, USA). In a Teflon jar, a quarter-gram sample (0.1000 g) was placed. Digestion was performed using 10 mL of Optima-grade Nitric acid (HNO_3_) and 3 mL of 30% Optima H_2_O_2_. Reconstituted samples were placed in 10 mL of 18.2 MΩ cm at 25 °C H_2_O with 2% nitric acid (both optima-grade). ICP-QMS quadrupole inductively coupled plasma-mass spectrometry was used to quantify metals (820 MS, Bruker). Calibration solutions were made from multielement standard stock solutions containing 20.00 mg/L of each element. Nine calibration solutions were used to generate calibration curves. In order to control reagent purity and laboratory equipment, reagent blanks containing ultra-pure water were also tested. A mix solution of internal standard (6 Li, 45 Sc, 72 Ge, 89 Y, 103 Rh, 159 Tb, 165 Ho, 209 Bi) of 10 µg/L, on-line aspired using a T union with the sample and standard solution, was used for determination. Every ten samples, a blank sample and at least two standard samples were tested to confirm the instrument calibration constants. A verified reference of NIST bovine liver was examined before and after each session of sample analysis to control the correctness of the analysis.

### 2.3. Exposure of Mussels to Nickel

To study the effects of nickel on *M. galloprovincialis*, specimens provided by Eurofish Napoli S.R.L. Bacoli (Campania region), with a medium shell size of 4.95 ± 0.17 cm and mixed sex, were selected. Mussels were exposed to different doses of NiCl_2_ (5, 15, and 35 μM) as reported in Piscopo et al., 2016 [48]. In brief, *M. galloprovincialis* specimens were housed in plastic tanks with a 36 × 22 × 22 cm size containing 6 L of 33‰ artificial sea water (ASW). Each L of ASW contained 29.2 g of NaCl, 0.60 g of KCl, 1.2 g of MgCl_2_, 0.20 g of NaHCO_3_, and 1.08 g of CaCl_2_. In each tank were placed 15 mussels for 24 h at 18 ± 1 °C. The oxygen and temperature levels of the tanks were checked periodically, and after 12 h, water and metal salts were exchanged. Experiments were carried out during February and March 2022. A tank containing only ASW was used as a control (unexposed mussels), as described by Lettieri et al., 2019 [45].

### 2.4. Processing and Sampling of Spermatozoa

After the exposure to 5, 15, and 35 μM NiCl_2_, mussels were opened with a knife. This operation was carried out leaving the soft tissues undamaged. The release of gametes was favored by leaving gonads at 16 °C for 5 min in 500 µL of ASW. Gametes were then observed under a microscope at 40× magnification in order to identify the sex of the specimens. Next, the gonads were left in the same tube for 1 h by adding another 500 µL of ASW in order to allow the release of all spermatozoa. To remove debris, the AWS containing spermatozoa was centrifuged at 2000× *g* for 1 min at 4 °C. The obtained supernatants were then centrifuged at 9000× *g* for 10 min, and the collected sperm pellets (of 200 µL) were stored at −80 °C.

### 2.5. Extraction and Analysis of PL Proteins from M. galloprovincialis Spermatozoa

For the extraction of protamine-like proteins from unexposed and Ni-exposed mussels, perchloric acid (PCA) was used at a final concentration of 5%, as described in [49]. For this experiment, *n* = 2 sperm pellets derived from mussels of each tank were used. The sperm pellets were added with 2 mL of distilled water and homogenized with a Teflon pestle. Subsequently, PCA was added. The acid extraction procedure was carried out as described by Vassalli et al., 2015 [46]. After 16 h at 4 °C on a shaking wheel, the samples were centrifuged at 14,000× *g* for 30 min, and the supernatants containing PCA-soluble PL proteins were extensively dialyzed against distilled water to remove PCA completely. Finally, a part of the dialysate was used immediately, while the remaining portion was stored at −80 °C.

### 2.6. Electrophoretic Analysis

Two types of electrophoretic analysis techniques were utilized to evaluate the pattern of the PL-proteins: AU-PAGE as previously described by Piscopo et al., 2018 [50] and SDS-PAGE as described below. The recipe for 14% polyacrylamide gel electrophoresis in urea-acetic acid (AU-PAGE) was as follows: 14% acrylamide/bis-acrylamide (starting with acrylamide/bis-acrylamide 25:0.67), 2.5 M urea, 5% acetic acid, 0.75% TEMED, and 0.15% APS. A 4–20% Tris-Glycine 1.0 mm gradient gel (Thermo Fisher, Waltham, MA USA) was used for SDS-PAGE. After AU-PAGE and SDS-PAGE, the gels were stained with Coomassie Brilliant Blue, and the image of the gels was acquired with a Gel-Doc system using ImageLab 6.0.1 (build 34) software (BioRad, Hercules, CA, USA). A densitometric analysis of the bands on the gel was carried out using the software ImageJ ver. 1.50 d (Wayne Rasband, National Institute of Health, Bethesda, ML, USA, https://imagej.nih.gov/ij/, 1997–2018) (accessed on 31 October 2022). Each experiment was repeated three times.

### 2.7. The Preparation and Analysis of Plasmid DNA

The plasmid DNA pGEM3 was isolated using ZymoPURE plasmid midiprep (D4200S) according to the companies’ instructions. The plasmid was purified from *Escherichia coli* HB 101 cells transformed with this plasmid according to the method described in Carbone et al., 2012 [51]. The quantification and purity of plasmid DNA were assessed with a UV-Vis instrument (NanoDropH ND-1000, Waltham, MA, USA), and, through 1% agarose gel electrophoresis in 89 mM Tris-HCl pH 8.0, 2 mM EDTA, and 89 mM boric acid (TBE), plasmid DNA integrity was evaluated.

### 2.8. Evaluation of the Effect of PL Proteins from M. galloprovincialis on the Electrophoretic Mobility of DNA

The effect of PL proteins of *M. galloprovincialis* after nickel exposure on the electrophoretic mobility of DNA was analyzed using the Electrophoretic Mobility Shift Assay (EMSA). The published procedure [50] was followed with some modifications. Specifically, to generate protein/DNA *w*/*w* ratios between 0.1 and 1.8, as shown in the results section, a fixed amount of plasmid DNA (pGEM3) (150 ng) and increasing amounts of PLs were utilized in all tests. The samples with a final volume of 30 µL were prepared with the following sequence: in the test tubes was placed ultrapure water (milliQ), DNA, and proteins. After that, the samples were kept at room temperature for 5 min to allow the proteins and DNA to interact. Following the interaction, TBE 10X and SB 10X were added to each sample at the final concentration 1X. The samples were analyzed by electrophoresis on 1% agarose gels at 100 V for 30 min. Gels were stained with ethidium bromide (2 mg/mL) after electrophoresis to visualize DNA and then acquired using a GelDoc system and ImageLab 6.0.1 (build 34), using the software BioRad, Hercules, CA, USA. Each experiment was repeated three times.

### 2.9. Preparation of Sperm Nuclei and Salt-Induced M.galloprovincialis Sperm Nuclear Basic Protein Release

For the preparation of the sperm nuclei, the procedure described in Olivares and Ruiz 1991 [52] was used. The release of sperm nuclear basic protein (SNBP) from sperm nuclei was achieved following the protocol reported in De Guglielmo et al., 2018 [53] by using the following increasing NaCl concentrations: 0.65 M, 0.8 M, 1.0 M, 2.0 M, 3.0 M, and 4.0 M. Sperm nuclei were gradually resuspended in 1 mL of NaCl solution at the indicated concentrations. For each suspension, incubation on a shaker for 30 min at 4 °C and then centrifugation for 30 min at 13,000× *g* were performed to obtain the supernatant containing the SNBPs that had been released at that salt concentration. The next salt solution was then added to the obtained pellet, and the procedure was repeated for all salt solutions. SNBPs were extracted with 0.2 N HCl (final concentration) from the supernatants obtained for each saline solution. The samples were incubated at 4 °C for 16 h and then centrifuged for 30 min at 13,000× *g*. The obtained supernatants were extensively dialyzed with distilled water. AU-PAGE of the obtained protein samples was performed in accordance with Fioretti et al., 2012 [44] by using 2.5 µg of each protein extract to determine the NaCl concentration required to produce the release of each PL protein from sperm DNA. For each different exposure condition, an extraction with 0.2 N HCl, directly from sperm nuclei, was performed, to obtain a sample containing total PL proteins and histones, to be used as a reference for quantifying protein bands in electrophoretic analysis. Each experiment was repeated three times.

### 2.10. Micrococcal Nuclease Digestion

Sperm nuclei obtained as described in the previous paragraph were resuspended in 1 mL of 0.15 M NaCl, 10 mM Tris—HCl, pH 8.0, 0.5 M CaCl_2_. Digestion with micrococcal nuclease (MNase) was conducted with 20 enzyme units (Sigma-Aldrich, Merk Life Science S.r.l., Milan, Italy) at 37 °C using a DNA concentration of 1 mg/mL (A260 = 20) and stopping the reaction at different times (5′, 15′, 30′, 60′) by adding 2 mM EDTA pH 8.0 and placing the sample in ice. The digests were then centrifuged at 1900× *g* for 10 min at 4 °C, and the resulting pellets were added with 1 M NaCl and 0.5% SDS and kept for 30 min at 25 °C. Then, a standard phenol/chloroform/isoamyl alcohol-based protocol was used to obtain DNA, which was analyzed on 0.9% agarose gels in TBE 1X at 60 V for 1 h. Each experiment was repeated three times.

### 2.11. Homogenates’ Preparation from Mytilus galloprovincialis Male Gonads

Homogenates’ preparation was performed according to Capriello et al., 2022 [54]. Gonad samples (0.3 g) were harvested, cut, and resuspended in 10 mM Tris-HCl pH 7.5, 1 mM EDTA, 1 mM EGTA, 0.15 mM spermine, 0.75 mM spermidine, 1 mM PMSF, 1 mM β-mercaptoethanol and 2 μg/mL, protease inhibitor cocktail (buffer A, 1:5 *w*/*v*). The tissue was homogenized for 15–30 s at low speed by an Ultra Turrax T8 (IKA Werke, Baden-Württemberg, Breisgau-Hochschwarzwald, Germany). All operations were carried out on ice or at 4 °C. The homogenate was centrifuged at 10,000× *g* for 15 min at 4 °C to remove cell debris. Separated supernatant fractions were collected for subsequent analysis [55]. Protein content was determined by Bradford’s reagent (BioRad, Hercules, CA, USA) according to De Maio et al., 2020 [56].

### 2.12. Western Blotting of PARP

Electrophoresis and the subsequent western blotting were performed according to Guerriero et al., 2018 [57]. Electrophoretic analyses of all the gonad homogenates (20 µg) were conducted on 12% polyacrylamide gels, and running was performed with the following buffer: 0.025 M Tris—0.192 M glycine—0.1% SDS at pH 8.3, at 18 mA. The gel was stained with 0.1% Coomassie Brilliant Blue R. For immunoblotting, electrophoresed proteins were transferred onto a polyvinylidene fluoride (PVDF) filter (0.45 µm; Cat No. IPVH00010, Merck Millipore, Milano, Italy) using a Bio-Rad Transblot system (BioRad, Hercules, CA, USA) at a constant 200 mA in 0.025 M Tris-0.192 M glycine—0.025% SDS buffer, pH 8.6, at 4 °C for 2 h. Immunochemical analysis of the blotted PVDF filter was performed with a monoclonal anti-poly(ADP-ribose)polymerase antibody (sc-8007, Santa Cruz Biotechnology, Inc., Dallas, TX, USA, 1:500) and horseradish peroxidase (HRP)-conjugated anti-mouse secondary antibodies (sc-525409, Santa Cruz Biotechnology, Inc., Dallas TX, USA, 1:2000). The HRP reaction was revealed by using a chemiluminescence’s kit (ECL Western Blotting Substrate, Pierce, Waltham, MA, USA), and the images were acquired by the ChemiDoc system (BioRad, Hercules, CA, USA). The immunopositive signal corresponding to the PARP-1 enzyme was quantified by densitometry analyses using the Image Lab software (BioRad, Hercules, CA, USA), and the densitometry was expressed as optical density (OD; i.e., intensity of a band/mm^2^). Each experiment was repeated three times.

### 2.13. Statistical Analysis

The intensity values of immunoreactive bands to anti-PARP were analyzed by the Kruskal–Wallis test and are shown as mean ± SD.

## 3. Results

### 3.1. Bioaccumulation of Nickel

**Figure 1:**
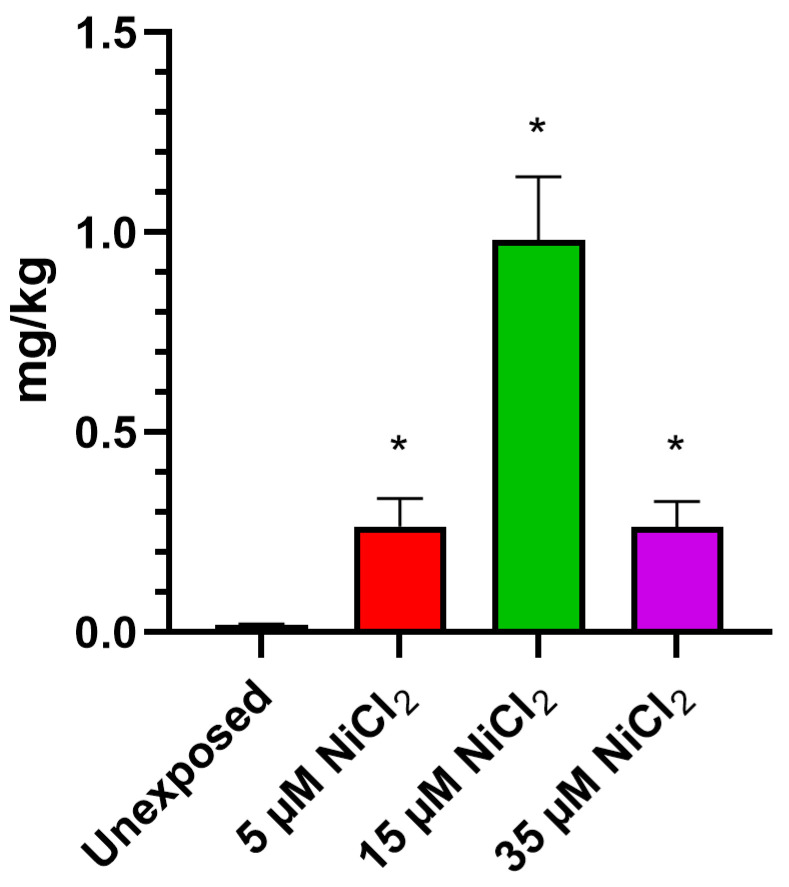
Evaluation of nickel bioaccumulation in gonad. *n* = 3. * = *p* < 0.05.

ICP-MS investigations demonstrate that exposing *M. galloprovincialis* to 5, 15, and 35 µM NiCl_2_ for 24 h produced bioaccumulation of this metal in gonadal tissue. The analysis showed an increase in nickel for all exposure conditions, with a highest value after the 15 µM NiCl_2_ exposure dose (Figure 1).

### 3.2. Electrophoretic Analyses of PL Proteins by AU-PAGE and SDS-PAGE

**Figure 2:**
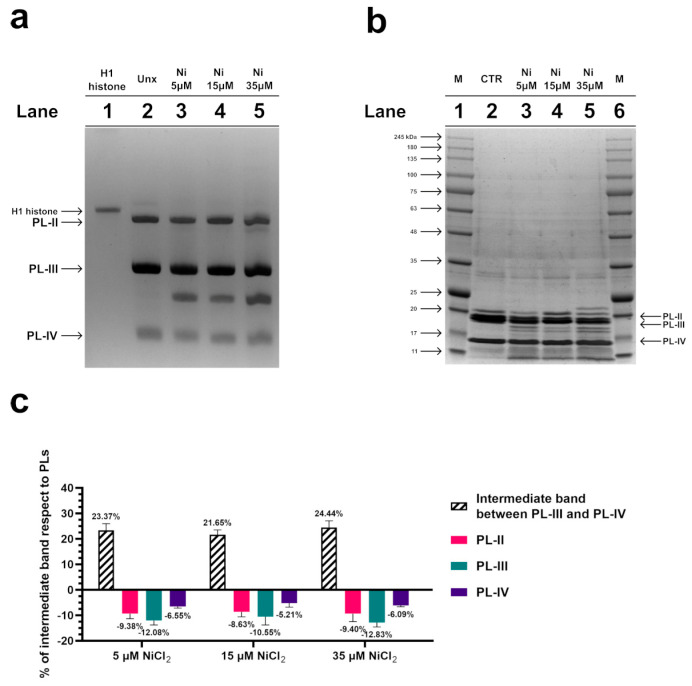
AU-PAGE (**a**) and SDS-PAGE (**b**) analyses of *M. galloprovincialis* PL proteins extracted from unexposed (Unx) (lane 2 of panels (**a**,**b**)) and exposed mussels (lanes 3–5 of panels (**a**,**b**)) to 5, 15, and 35 µM NiCl_2_, respectively. Lane 1 of AU-PAGE shows sperm H1 histone from the annelid worm *Chaetopterus variopedatus*. M in SDS-PAGE (panel (**b**), lanes 1 and 6) denotes molecular weight marker. (**c**) evaluation of the % of intermediate band respect to PLs, by densitometric analysis. The intermediate band is the additional protein band migrating between PL-III and PL-IV, visible in AU-PAGE. *n* = 3.

To investigate possible alterations in the electrophoretic pattern of PL proteins after exposure of mussels to 5, 15, and 35 µM NiCl_2_, an AU-PAGE was conducted. This type of analysis showed an additional band migrating between PL-III and PL-IV following all exposure doses (Figure 2a, lanes 3–5), which was absent in the unexposed condition. The densitometric analysis shown in the panel c indicated that this additional protein band, intermediate between PL-III and PL-IV, derived from the degradation of a fraction of all PL proteins. SDS-PAGE analysis confirmed this result, revealing that a fraction of the PL proteins’ samples was in the form of peptides with different molecular weights, indicative of hydrolysis of a fraction of PL proteins following exposure of the mussels to these three doses of NiCl_2_ (Figure 2b).

### 3.3. EMSA

**Figure 3:**
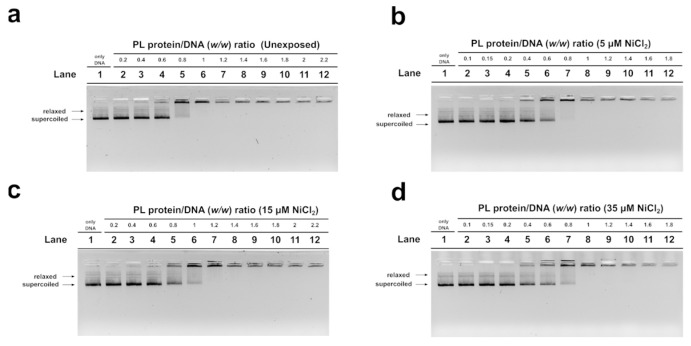
Analysis of the ability of PL proteins to bind DNA from unexposed (**a**) and exposed mussels to 5, 15, and 35 µM NiCl_2_ ((**b**), (**c**), and (**d**), respectively) by EMSA, using pGEM3 plasmid DNA. Numbers on the wells indicate PL protein/DNA (*w*/*w*) ratios used; only DNA indicates pGEM3 plasmid DNA without added protein. *n* = 3. Relaxed and supercoiled are the two topological forms of pGEM3 plasmid DNA.

In order to evaluate the DNA binding ability of PL proteins, EMSA was performed using the pGEM3 DNA plasmid as a probe, as reported in Vassalli et al., 2015 [46]. In each experimental condition, the protein-to-DNA (*w*/*w*) ratio required to achieve DNA saturation, that is, when all plasmid DNA was close to the well, was evaluated. For the unexposed mussel, the DNA saturation was achieved at a PL proteins/DNA ratio of 1.0 (Figure 3a, lane 6) as well as in the exposure conditions of 35 µM NiCl_2_ (Figure 3d, lane 8). After 15 µM NiCl_2_ exposure, on the other hand, DNA saturation required a little more of the PL proteins, specifically a PL protein/DNA ratio of 1.2 (Figure 3c, lane 7). Finally, after 5 µM NiCl_2_ exposure, DNA saturation required fewer PL proteins, precisely a PL protein/DNA ratio of 0.8 (Figure 3b, lane 7).

### 3.4. Release of PL Proteins from Sperm Nuclei

**Figure 4:**
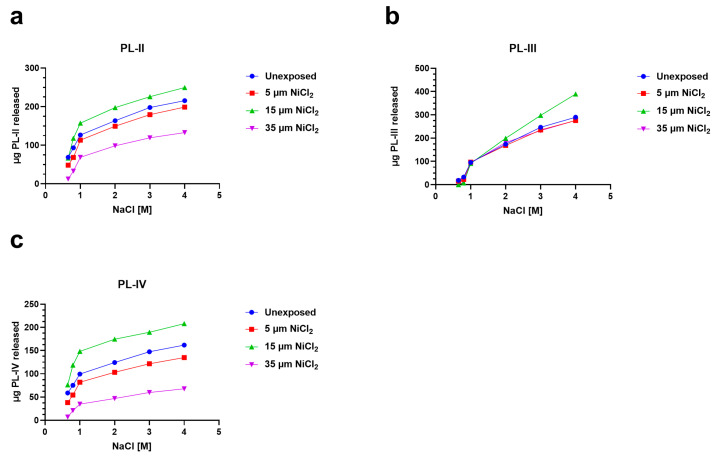
Release of PL proteins from sperm nuclei with increasing molar concentrations of NaCl in the unexposed mussels and exposed to 5, 15, and 35 µM NiCl_2_. The data are presented as mean ± S.D. *n* = 3. (**a**): PL-II; (**b**): PL-III and (**c**): PL-IV.

To investigate the possibility of alterations in PL proteins-DNA binding, after the exposure of mussels to nickel, we analyzed the release of PL proteins with increasing concentrations of NaCl from sperm nuclei in unexposed and exposed mussels. In particular, for the 5 and 35 µM NiCl_2_ conditions, the release of PL-II and PL-III was lower than the unexposed condition (red and violet lines, Figure 4a,b). Exposure to 15 µM NiCl_2_, on the other hand, always produced an increase in PL protein release compared with the unexposed condition (green lines Figure 4a–c).

### 3.5. MNase Digestion Pattern of M. galloprovincialis Sperm Chromatin

**Figure 5:**
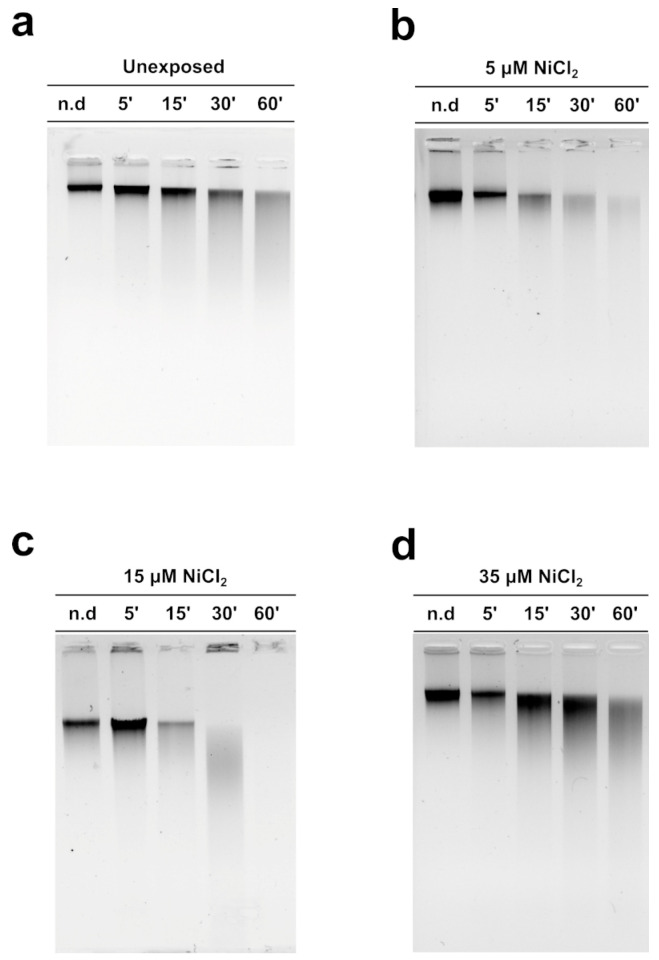
MNase digestion time course of *M. galloprovincialis* sperm chromatin. Analyses of DNA by electrophoresis on 0.9% agarose gel: unexposed (panel (**a**)) and exposed mussels to 5, 15, and 35 µM (panels (**b**), (**c**), and (**d**), respectively). n.d. = Sperm DNA not digested; *n* = 3.

Figure 5 shows the MNase digestion time course of *M. galloprovincialis* sperm chromatin for 5, 15, 30, and 60 min. The analyses were conducted on sperm chromatin of unexposed and exposed mussels to the three NiCl_2_ doses. The DNA contained in the fractions produced at different MNase digestion times was analyzed on agarose gel. The typical electrophoretic pattern of sperm chromatin of this organism after MNase digestion was obtained in the unexposed condition (Figure 5a). Following mussels’ exposure to 5 and 15 µM NiCl_2_, but in particular at the 15 µM NiCl_2_ dose, a higher accessibility of MNase to the sperm DNA was observed (Figure 5b,c), indicative of an improper sperm chromatin structure. The exposure dose of 35 µM NiCl_2_, on the other hand, produced a result very similar to that obtained in the unexposed condition (Figure 5d).

### 3.6. PARP Expression

PARP expression analysis was conducted on gonad homogenates of *M. galloprovincialis* exposed to the three nickel concentrations (5, 15, and 35 µM NiCl_2_) and in mussels not exposed to nickel.

**Figure 6:**
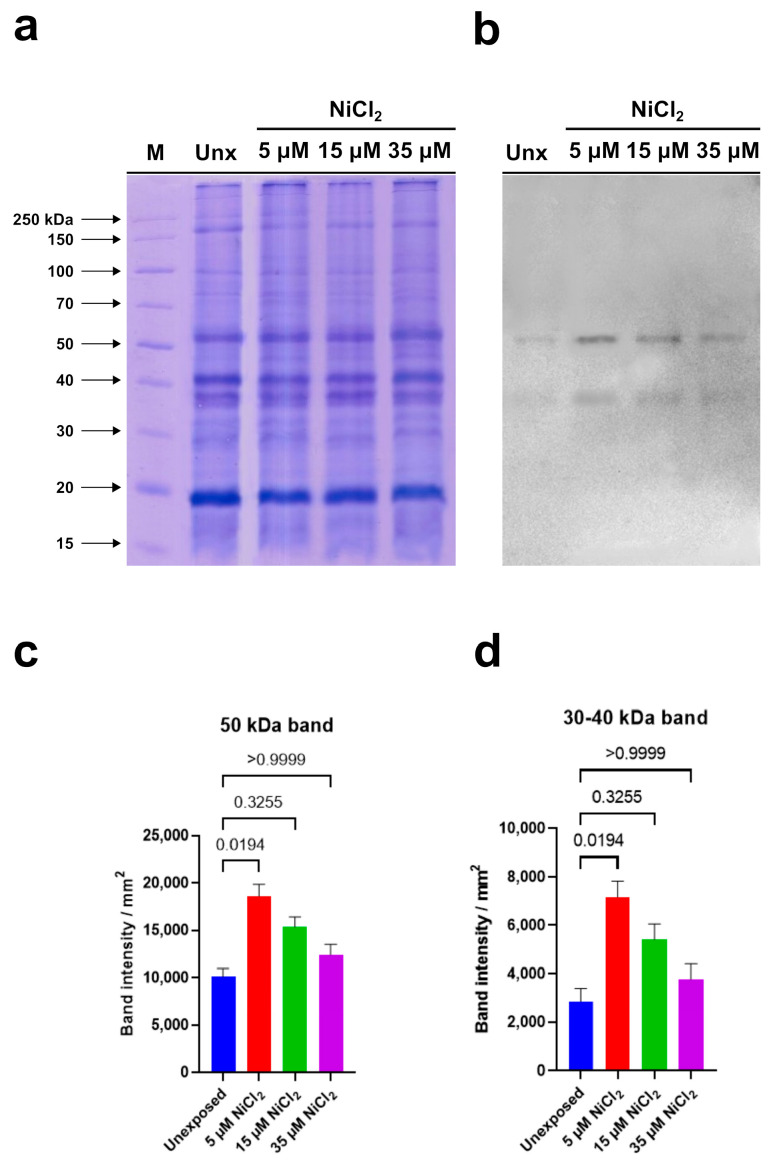
Western blotting carried out in homogenates of *M. galloprovincialis* gonads of unexposed and exposed mussels to 5, 15, and 35 µM NiCl_2_. (**a**): 12% Sodium dodecyl sulfate polyacrylamide gel electrophoresis (SDS-PAGE). (**b**): anti-PARP immunoblotting. (**c**,**d**): densitometric analysis. The histograms represent mean ± SD. Results were analyzed by Kruskal–Wallis’ test: the intensity of 50 kDa band (**a**) and that between 30–40 kDa (**b**) in samples of exposed mussels to 5 and 15 µM NiCl_2_ was significantly higher than that determined in the unexposed condition. *n* = 3.

Electrophoretic analysis showed no significant qualitative and quantitative differences in the protein patterns of all examined samples (Figure 6a).

Immunoblotting, performed using the anti-PARP antibody, able to recognize the highly conserved catalytic site of PARP-1, evidenced two immunoreactive bands: the first corresponding to a protein of 50 kDa and the second to a protein with a molecular weight between 30 and 40 kDa (Figure 6b).

Densitometric analysis of immunoreactive bands showed that the intensity of the signal corresponding to the protein with a molecular weight between 30 and 40 kDa is always lower than that measured in correspondence of the 50 kDa protein in the unexposed condition and in the samples deriving from mussels exposed to nickel. In addition, a significant intensity increase in both immunopositive signals was observed in homogenates of gonads from mussels exposed to 5 µM and 15 µM NiCl_2_ compared to unexposed mussels. In detail, the highest intensity was measured following 5 µM NiCl_2_ exposure. Higher nickel concentrations (35 µM) did not produce significant intensity variations in both immunoreactive signals compared to unexposed condition (Figure 6c,d). In Table 1 is shown the multiple comparison of the Kruskal–Wallis’ test.

## 4. Discussion

Nowadays, technological and industrial progress leads to more and more substances in the environment, and for this reason, a variety of biomonitoring systems have been developed [58,59,60]. Nickel is a metal extensively distributed in the environment. In fact, it derives from natural sources and anthropogenic activity. Exposure to nickel can cause a wide range of side effects including those on the reproductive health of marine and terrestrial organisms, including humans [31]. Although the negative effects of this metal on the male reproductive sphere have been widely demonstrated, being the subject of three reviews in the last decade [61,62], the molecular mechanisms of nickel-induced toxicity on reproduction are still not fully understood. To obtain a clearer idea of the possible molecular mechanisms underlying nickel reproductive toxicity, we proposed to study the effects of this metal in *M. galloprovincialis,* a very useful organism as a bioindicator and bioaccumulator. After exposure of *M. galloprovincialis* specimens to 5, 15, and 35 µM NiCl_2_, we found accumulation of nickel in the gonad of this organism at all exposure doses but in particular at 15 µM NiCl_2_. This result prompted us to investigate possible alterations in the properties of *M. galloprovincialis* PL-proteins, the latter being the main component of the sperm nuclear basic proteins of this organism. In fact, PL-proteins play a key role in the structuring of sperm chromatin. The electrophoretic analysis of these proteins showed, by AU-PAGE, the presence of a protein band with an intermediate mobility between PL-III and PL-IV in the spermatozoa of mussels exposed to all doses of NiCl_2_, differently to unexposed mussels. This protein band corresponded to various peptides, as demonstrated by SDS-PAGE, and indicated that mussels’ exposure to these doses of NiCl_2_ caused the hydrolysis of a fraction of these proteins. This result is in accordance with data in the literature which indicate that nickel can bind to specific amino acids, such as serine and threonine, and cause hydrolysis of the peptide bond [63]. As a matter of fact, PL proteins, are extremely rich in serine, and then nickel could cause the formation of peptides from these proteins. Moreover, the strong tendency of basic peptides to self-associate was also reported [64,65]. Concerning the DNA binding of PL proteins from exposed mussels, we found that, under all experimental conditions, these proteins interacted with DNA in an all-or-nothing mode, as already reported for histone H1 [43,66]. Furthermore, differences in DNA-binding affinity were observed following exposure to nickel. Specifically, exposure of mussels to 5 µM NiCl_2_ slightly increased the DNA-binding ability of PL proteins compared with the unexposed condition, while exposure to 15 µM NiCl_2_ reduced their DNA-binding ability. These differences in the DNA binding of PL proteins also influenced the release of these proteins from the sperm nuclei. The highest release of PL-II and PL-III occurs at 15 µM NiCl_2_, while the lowest at 35 µM NiCl_2_ compared to the unexposed condition. This evidence suggests that the higher or lower DNA binding ability of PLs predicts their lower or higher release from sperm nuclei, respectively [5,67]. Differences in the binding of PL proteins to DNA may affect the structure of sperm chromatin, the degree of compactness which is known to be critical for the ability of spermatozoa to swim and consequently their fertilization potential. The evidence that at all doses of NiCl_2_ exposure, there was increased accessibility of micrococcal nuclease to sperm chromatin may be indicative of improper organization of sperm chromatin, probably more decondensed, a condition particularly relevant after the 15 µM exposure dose of NiCl_2_, a dose at which the highest value of nickel accumulated in the gonad was also found. Therefore, nickel can also cause changes in PL protein properties as our group already demonstrated for other metals, such as mercury [5,36,37], cadmium [53], copper [7], and chromium [67]. Finally, the exposure to these doses of NiCl_2_ also seems to influence the expression of the two PARP isoforms identified by western blotting in gonad homogenates of *M. galloprovincialis*. In detail, we suppose that the expression of these enzymes might be related to chromatin compaction. On the other hand, it is well known that mammalian PARP1 is a nuclear enzyme involved in both structural and regulatory roles across the genome. It is considered a “sensor” of DNA damages and is also involved in the regulation of chromatin structure and genomic integrity [68]. 

The evidence in mussels that both the 50 kDa PARP and that with molecular weights between 30 and 40 kDa are more expressed in samples exposed to 5 µM NiCl_2_ than in the unexposed condition leads us to suppose that this dose is already sufficient to induce DNA damages. In addition, at this same dose, a greater PARPs synthesis would be required because the chromatin compaction obtained at this exposure dose would not yet allow the access of the repairing enzymes to the damage sites.

**Figure 7:**
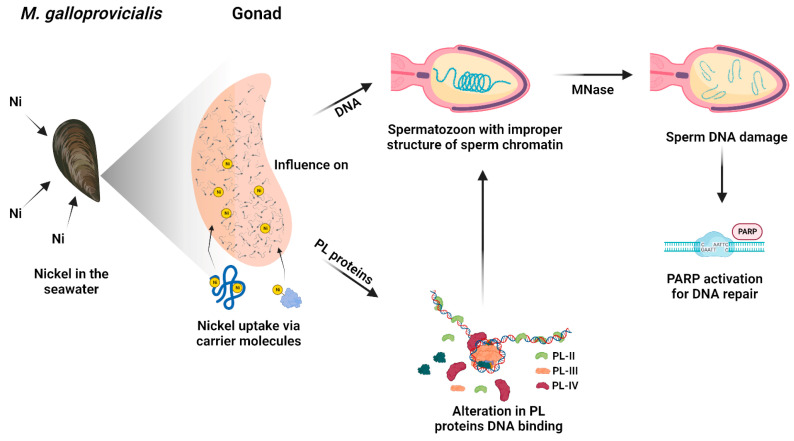
Hypothetical molecular model that explains nickel-induced reproductive toxicity in *M. galloprovincialis*.

On the contrary, in the samples exposed to 15 µM NiCl_2_, the expression of PARP does not increase significantly compared to the unexposed condition, since the intervention of the repairing enzymes would be facilitated by the more relaxed structure of the chromatin. The lower and higher accessibility of the repair enzymes to the DNA damage sites at 5 and 15 µM NiCl_2_, respectively, is also confirmed by MNase data. That the sperm chromatin structure in mussels exposed to nickel is more susceptible to the action of micrococcal nuclease is in line with what has been reported on the effects of nickel in mammals. Indeed, it is well known that, in mammals, in the process of spermatogenesis, the histones are gradually substituted by protamines to package DNA efficiently. Nickel is recognized as interfering with the interaction between protamines and DNA via affecting the protamine functional structure. In conclusion, all these data show that although PL proteins are extremely important proteins for the correct compaction of sperm chromatin, they are very susceptible to external factors [35,36,37,50] and consequently can affect the correct packaging of chromatin and the fertilizing capacity of spermatozoa. Taking into account all the results of this work, we propose a hypothetical molecular model that explains nickel-induced reproductive toxicity in *M. galloprovincialis* (Figure 7).

In detail, nickel present in seawater can enter in the mussels by filtration and, through carrier molecules, can reach mussels’ tissues including gonads. Although they are not tissues used for filtration and digestion, gonads are demonstrated to have a similar accumulation ability of metals with respect to gills and digestive glands [7]. In addition, the metal accumulated in sperm and PL proteins is generally very similar, indicating that the metal accumulating in PL proteins is responsible for the metal accumulated in spermatozoa [7]. Nickel can affect PL proteins’ properties which in turn impact sperm DNA. Nickel could produce conformational changes in PL proteins as demonstrated in our previous works for copper [7], cadmium [53], Hg [5,36,37], and chromium [67]. The conformational changes in PL proteins alter their DNA binding and in turn affect sperm chromatin structure. The probably improper structure of sperm chromatin in the spermatozoa of mussels exposed to nickel, but particularly in those exposed to 15 µM NiCl_2_, might be more decondensed with respect to the unexposed condition and thus more accessible to MNase. In fact, at all doses of nickel exposure, sperm DNA damage is observed at low MNase digestion times, but particularly at the exposure dose of 15 µM NiCl_2_. Damage to sperm DNA, which already occurs at the exposure dose of 5 µM NiCl_2_, induces PARP activity. *M. galloprovincialis* PL-proteins belonging to the group of SNBP could be a “target” of nickel attack as that which occurs for protamine 2 (P2), which is essential for sperm production and maturation in mammalian cells. This can be achieved because of the particular structure of protamine 2 that makes its binding to zinc possible. Nevertheless, it is also possible to replace zinc with other metal ions, e.g., Ni(II) [69]. The nickel-protamine P2 interaction has been found to inhibit normal chromatin condensation. Ni(II) oxidative activity increases its interaction with P2 and results in changes in DNA structure and the formation of oxidation products, which are promutagens [70]. These findings emphasized the importance of studying the toxicity of nickel on the reproduction of organisms, taking into account that several research studies in mammals suggested that late-stage germ cells are less tolerant to ROS than early-stage germ cells, principally because of their limited reserve of antioxidant enzymes. The reason for this is that zinc levels decrease during spermatogenesis. In fact, zinc has a significant role as a DNA stabilizer, being essential for several DNA repair enzymes that are important during early embryogenesis [71,72] and modulates the activity of SOD [73]. In conclusion, given the changes found in the properties of PL proteins following exposure to nickel in *M. galloprovincialis*, it would be interesting in the future to evaluate any conformational changes in these proteins further by fluorescence spectroscopy analysis and circular dichroism, but, to determine with more certainty whether these doses of nickel can cause impairments in the fecundation potential of spermatozoa, in vitro fertilization experiments will be planned. 

## Figures and Tables

**Table 1:** Results of multiple comparison of Kruskal–Wallis’ test.

Dunn’s Multiple Comparisons Test	Significant?	Summary	Adjusted *p* Value
Unexposed vs. 5 µM NiCl_2_	Yes	*	0.0194
Unexposed vs. 15 µM NiCl_2_	No	ns	0.3255
Unexposed vs. 35 µM NiCl_2_	No	ns	>0.9999
5 µM NiCl_2_ vs. 15 µM NiCl_2_	No	ns	>0.9999
5 µM NiCl_2_ vs. 35 µM NiCl_2_	No	ns	0.1887
15 µM NiCl_2_ vs. 35 µM NiCl_2_	No	ns	>0.9999

* = *p* < 0.05.

## Data Availability

Not applicable.
